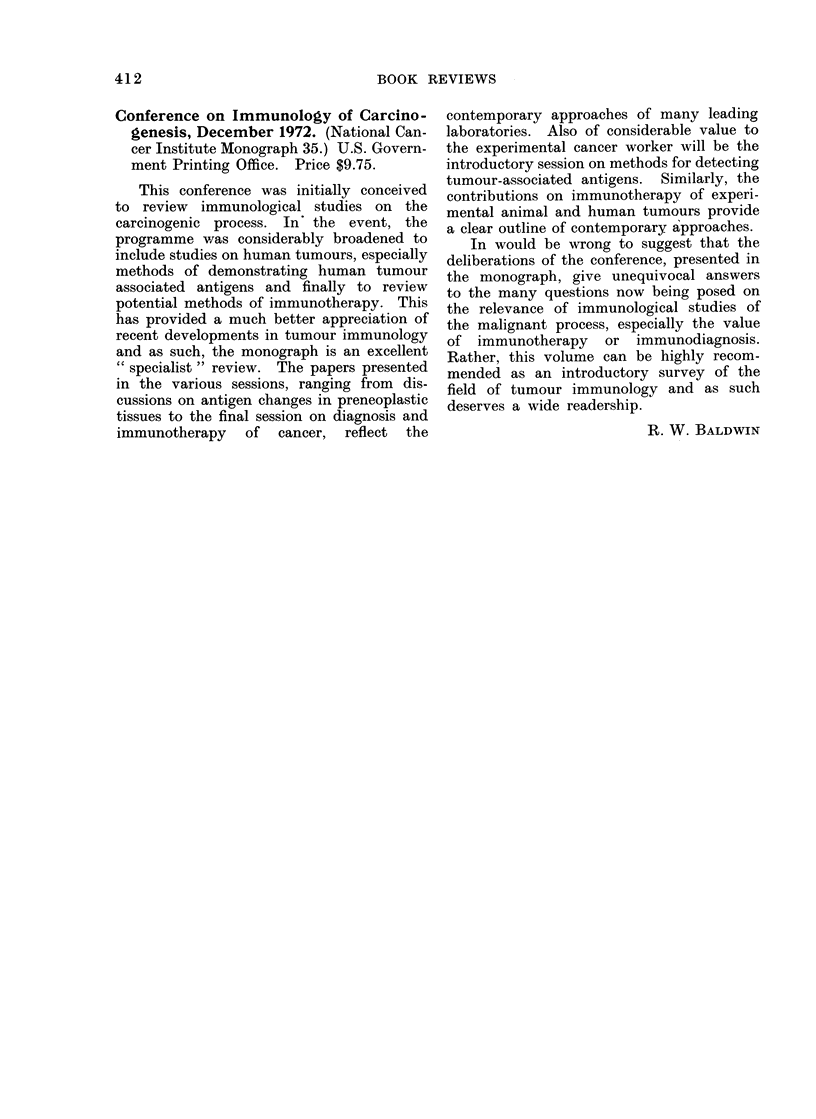# Conference on Immunology of Carcinogenesis, December 1972

**Published:** 1974-05

**Authors:** R. W. Baldwin


**Conference on Immunology of Carcinogenesis, December 1972.** (National Cancer Institute Monograph 35.) U.S. Government Printing Office. Price $9.75.

This conference was initially conceived to review immunological studies on the carcinogenic process. In the event, the programme was considerably broadened to include studies on human tumours, especially methods of demonstrating human tumour associated antigens and finally to review potential methods of immunotherapy. This has provided a much better appreciation of recent developments in tumour immunology and as such, the monograph is an excellent " specialist " review. The papers presented in the various sessions, ranging from discussions on antigen changes in preneoplastic tissues to the final session on diagnosis and immunotherapy of cancer, reflect the contemporary approaches of many leading laboratories. Also of considerable value to the experimental cancer worker will be the introductory session on methods for detecting tumour-associated antigens. Similarly, the contributions on immunotherapy of experimental animal and human tumours provide a clear outline of contemporary approaches.

In would be wrong to suggest that the deliberations of the conference, presented in the monograph, give unequivocal answers to the many questions now being posed on the relevance of immunological studies of the malignant process, especially the value of immunotherapy or immunodiagnosis. Rather, this volume can be highly recommended as an introductory survey of the field of tumour immunology and as such deserves a wide readership.

R. W. Baldwin